# Progress and Applications of Nanocomposites in the Technology of Biosensors

**DOI:** 10.3390/nano15241905

**Published:** 2025-12-18

**Authors:** Catalina Cioates Negut, Raluca-Ioana Stefan-van Staden, Ruxandra-Maria Ilie-Mihai

**Affiliations:** Laboratory of Electrochemistry and PATLAB, National Institute of Research for Electrochemistry and Condensed Matter, 202 Splaiul Independentei Str., 060021 Bucharest, Romania; negutcatalina79@gmail.com

**Keywords:** nanocomposite, biosensor, healthcare, environment

## Abstract

There has been tremendous progress in the development and application of nanotechnology in the past ten years. There are a plethora of nanoparticles and nanomaterials that have been developed and used to improve the biosensors’ overall performance. Nanocomposites integrate several nanomaterials inside a matrix to improve their structural and functional characteristics, resulting in enhanced biosensor efficacy. This review covers the achievements in nanocomposites containing metal, polymer, inorganic, carbon-based, or gold nanoparticles as new biosensors for detecting a wide range of (bio)molecules with improved sensitivity, selectivity, and a low limit of detection. The purpose is to give an overview of current advances and applications in the field of nanocomposites utilized in biosensors’ design. Emphasis will be placed on the possible uses of these nanocomposites in biosensing across a range of industries, medication delivery, food safety, healthcare, and environmental monitoring.

## 1. Introduction

Nanotechnology is very often used in health and many other technologies. There is a wide variety of uses for nanomaterials, which are composed of extremely tiny particles with well-defined dimensions [1]. The incorporation of nanotechnology into everyday operations improves our quality of life because of the special characteristics and functions that these materials provide. Both the qualitative and quantitative elements, as well as its financial and technological impacts, are substantially amplified through the incorporation of the various alternatives provided by nanoscience [2].

**Figure 1:**
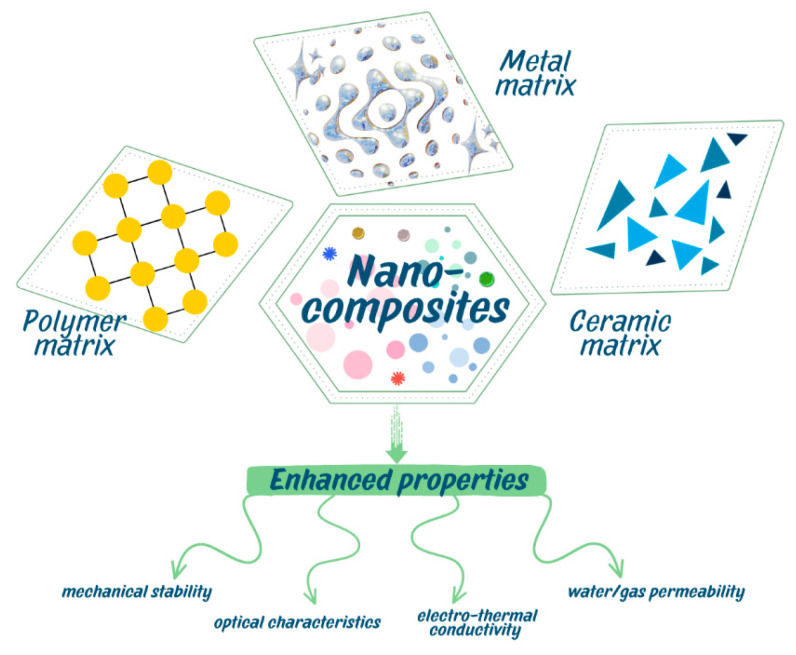
Types of nanocomposites classified accordingly to the matrix used.

Nanocomposites are favored in both academic and industrial spheres owing to their exceptional features, distinctive design potential, environmentally benign characteristics, straightforward manufacture, and economic viability. The integration of nanoparticles (NPs) into a matrix of specific materials (such as polymers, metals, or ceramics) enhances their distinctive properties, including superior mechanical stability (in terms of strength, toughness, flexibility, Young’s modulus, and dimensional stability), favorable optical characteristics, flame retardancy, low water/gas permeability, and high electro-thermal conductivity [3]. As shown in Figure 1, nanocomposites may therefore be composed of a variety of matrix composites, including metal [4], polymer [5], and ceramic [6].

Nanocomposites are mixes of several materials and phases that include at least one nanostructured material, such as NPs. Enhanced performance of such sensors has been achieved as a consequence of the exceptional characteristics of nanocomposites, which include their large surface area, high mechanical stability, and electrical conductivity. This has led to the development of a family of biosensors that is both advantageous and stable. Many different nanocomposites have been used in this application for a variety of goals, including the construction of fast-responding sensors as well as the increase in the overall sensors’ sensitivity and selectivity. Graphene, metal oxides, and carbon nanotubes (CNTs) are some examples of the nanostructure fillers that are used in the production of nanocomposites. The application of these materials in biosensing has led to the emergence of numerous beneficial characteristics and properties. We will emphasize the significance of nanocomposites and their associated nanostructured materials in the advancement of biosensors. Ultimately, we will highlight the advantages derived from employing diverse nanocomposites in biosensors.

**Figure 2:**
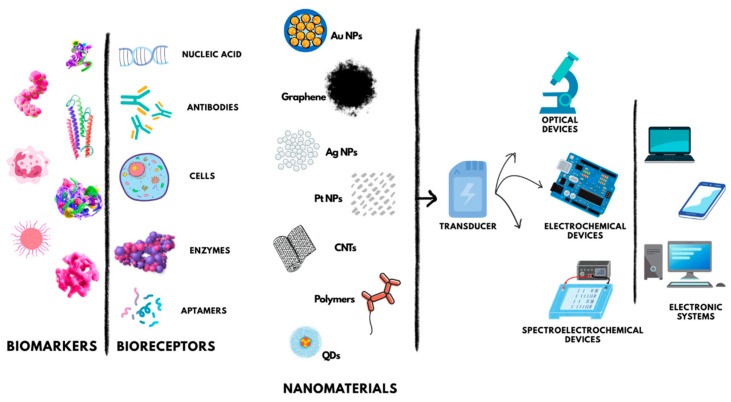
Schematic illustration of biosensors.

Biosensors are analytical tools that measure certain compounds by combining a biological component with a physicochemical detector. These gadgets translate biological processes into electrical or optical impulses that may be measured (Figure 2).

In 1962, Clark and Lyons initially invented biosensors by employing an oxygen electrode to selectively detect glucose levels [7].

Currently, biosensors are extensively utilized in biomedicine, health, environmental monitoring, drug research, forensics, and assurance of food quality due to their elevated sensitivity, specificity, and rapid response times. They are essential tools in contemporary technology for effectively detecting a variety of biochemical changes because they combine biological and electrical components. Biosensors are essential for identifying and measuring analytes as glucose, lactate, electrolytes, and other biomarkers in biological fluid analysis.

For the non-invasive, continuous tracking of disease-indicating biological markers in biofluids, for example, perspiration, urine, saliva, tears, and breath, electrochemical biosensors have been developed. These sensors are attractive instruments for personalized health monitoring because of their great sensitivity, selectivity, and affordability [8]. By offering quick, quantifiable diagnostic data without requiring centralized laboratory procedures, the incorporation of biosensors into point-of-care (POC) settings has the potential to drastically alter patient care. This technique may yield improved patient outcomes and reduce load on the medical system [9]. The emphasis on developing biomarkers is motivated by the necessity of early disease detection and surveillance. To enable earlier interventions and individualized treatment plans, emerging biomarkers can offer vital information regarding disease states. These indicators may now be found in bodily fluids with greater sensitivity, specificity, and speed thanks to microfluidic biosensors.

With a primary focus on research applications of nanocomposites in the field of biosensors, this review is based on a systematic literature search utilizing the Web of Science Core Collection database and Google Scholar. The primary emphasis of the search was research applications of nanocomposites in the biosensors sector. The three types of nanocomposites that were included in the search were metal, polymer, inorganic, carbon-based, and gold nanoparticles (AuNPs). This review contains articles published in the last decade in order to ensure that the research encompassed the evolution of this field, which is changing at an incredibly fast pace. After the scientific papers were collected, they were subsequently examined in order to verify that they were pertinent to the objectives of the review. Studies and articles not written in English and those that did not concern nanocomposites with metal, polymer, inorganic, carbon-based or (AuNPs) were not considered.

## 2. Nanocomposite Used in Biosensors

It is possible for nanocomposites to include composites that can be made using metal matrices [4], polymers [5], or ceramics [6].

### 2.1. Metal Nanocomposites Used in Biosensors

Metal nanoparticles (MNP) are integrated with additional substances, like polymers or ceramics, to synthesize metal nanocomposites with improved or distinctive properties. These materials provide distinctive combinations of mechanical, electrical, optical, and thermal capabilities by combining the beneficial qualities of their constituent parts [10]. Metal-polymer nanocomposites are interesting prospects for conductive filler and coating applications because they combine the flexibility and superior manufacturability of polymers with the superior plasmonic, electrical, and thermal properties of metals [11]. Metal nanocomposites provide significant advantages in biosensing, including an increased activity as a catalyst in addition to greater electrical conductivity, which can raise the biosensors’ sensitivity and selectivity. For instance, adding MNP to a polymer matrix can result in a nanocomposite with specialized electrical characteristics that can be used to detect particular biological analytes [12]. Biosensors are enhanced by metal nanocomposites thanks to five main benefits. Through plasmonic effects and a high surface area, a detection limit enhancement of up to 1000 times is possible with the use of AuNPs or silver nanoparticles (AgNPs), improving sensitivity [13], multimodal sensing, which allows for optical, electrochemical, and magnetic detection on a single platform, and accurate targeting. Nanoparticles with surface modifications bind to biomarkers specifically while avoiding interference [14]. Because of their increased endurance, metallic nanocomposites can endure challenging circumstances and continue to function steadily over time [15]. Additionally, they aid in the creation of small, flexible biosensors that are perfect for applications involving wearable health monitoring [16]. These developments are revolutionizing environmental monitoring and medical diagnostics, particularly for POC applications where sensitivity and dependability are crucial. A system for analyzing perspiration and detecting potential infectious diseases are recent innovations that show the technology’s potential for practical use. The detection of new biomarkers in biological fluids can benefit greatly from the integration of microfluidic technology into biosensors. Specimens that undergo sorting or processing through the use of microfluidic devices can have their biomarker composition and concentration measured using microfluidic biosensors [17].

Many studies have focused on metals and metal-oxide composites due to their malleability and the fact that they can be used to make medical devices with high detection accuracy. The strength, conductivity, and heat resistance of polymers are generally limited. Metal–polymer nanocomposites with enhanced optical and electrical characteristics were created to broaden their applications [18].

Because of their unique characteristics, MNP—also referred to as plasmonic NPs [19]—are employed in fluid detection. They are regarded as exceptional optical substitutes; because of their electrical, mechanical, and magnetic characteristics, they can be used to fabricate electronic devices. Noble metals, such as silver (Ag) [20], gold (Au) [21], palladium (Pd) [22], or platinum (Pt) [23] in conjunction with nanotechnology [24], offer a promising approach to creating intelligent instruments to treat medical issues. Because of their versatility, superior optical and catalytic qualities, and potential as an effective target in specific applications, Ag and Au are regarded as the best options for creating nanomaterials [25]. Furthermore, their primary benefit is their plasmonic activity [26], which promotes their employment as photonics in fluid-detecting biosensor applications.

**Figure 3:**
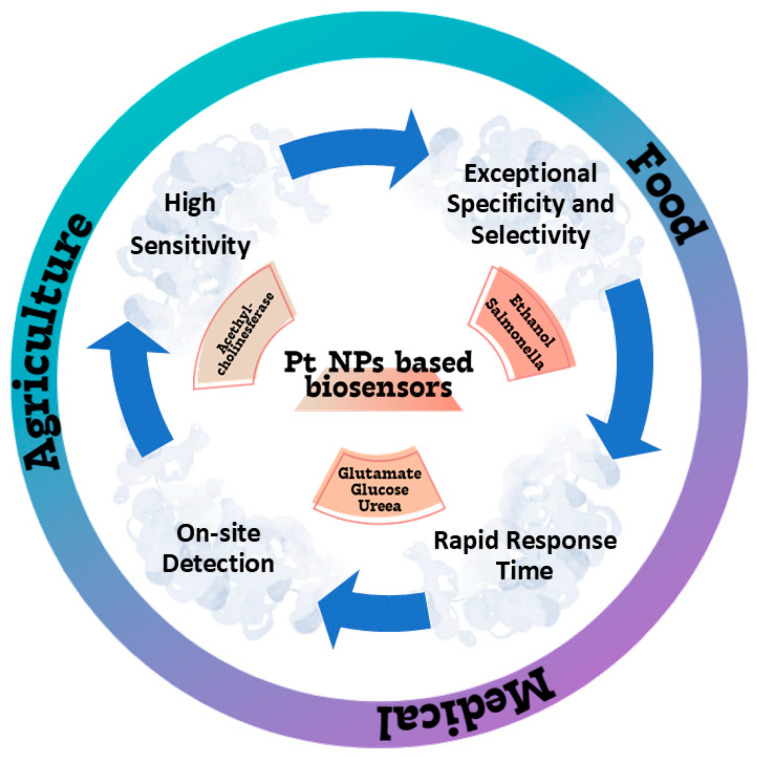
Applications of Pt-based biosensors in food, agricultural, and medical areas.

The food, agricultural, and medical areas were identified as prospective uses for Pt nanoparticle-based biosensors (Figure 3).

The applications of platinum nanoparticles (PtNPs)-based biosensors in the food industry include the glassy carbon electrode (GCE)/mixed manganese and molybdenum oxides/Pt, an effective biosensor for ethanol in alcoholic beverages with detection range of 0.075–5 mM, with a response time of 63 s [27]; magnetic beads coated with Pt and Pd nanoparticles paired with DNA aptamers were utilized in the biological analysis carried out by Dehghani et al. We used it in conjunction with loop-mediated isothermal amplification to detect Salmonella Typhi-murium in both food and stool samples. Even at concentrations as low as 10–15 and 3–10 CFU mL^−1^ in egg and chicken fecal specimens, respectively, the as-say was able to accurately identify Salmonella Typhimurium in less than three hours. A relative accuracy of 90% was attained, with intra-assay and inter-assay precision of 8.36% and 9.92%, respectively, in the Salmonella Typhimurium-spiked food samples. This devised approach has promise for incorporation into lab-on-a-chip biosensors, alongside pre-chip pathogen concentration, for the on-site detection of Salmonella Typhimurium and other pathogens in diverse food and non-food samples [28].

Numerous biosensor varieties are founded on PtNP. Besides PtNP, there are sensors which utilize Pt oxides and Pt alloys, where they could one day find application in real-life situations [29,30].

Platinum nanoparticle-based biosensors are successfully used in the farm sector. Alternative tools, like acetylcholinesterase (AChE) amperometric biosensors, exhibit heightened sensitivity, accessibility, and cost-effectiveness [31,32] in comparison to prior environmental monitoring techniques. Ma et al. created Pt@UiO66–NH_2_ nanocomposites by combining zirconium-based organic framework nanomaterials (UiO66–NH_2_) with chloroplatinic acid, deionized water, and ascorbic acid. The Pt@UiO66-NH_2_ nanocomposites are crucial for minimizing noble metal usage while offering an exceptionally large surface area and increased adsorption sites, hence creating a conducive environment for immobilized AChE. The developed biosensor is capable of detecting residues of organophosphorus pesticides in the environment. The MNP/ metal–organic framework (MOF) nanocomposites may present novel options for the advanced biosensor and facilitate broader applications [33].

Platinum nanoparticle-based biosensors are significant in the medical domain. This method may efficiently and swiftly identify many chemical constituents in bodily fluids, including glutamic acid, glucose, and urea.

#### 2.1.1. Glutamate Detection

Dalkıran et al. devised an innovative amperometric biosensor utilizing a graphene/tricobalt tetraoxide nanoparticles/chitosan (GR/Co_3_O_4_/CS) nanocomposite for the detection of glutamate. The incorporation of GR and Co_3_O_4_ nanoparticles enhanced the analytical performance of the glutamate biosensor, evidenced by a reduced reaction time (25 s), an extensive linear range (4.0 × 10^−6^–6.0 × 10^−4^ M), excellent repeatability (1.0%), suitable stability, and immunity to common interfering chemicals. Furthermore, the expense associated with the proposed biosensor is inferior to that of traditional methods, and its fabrication process is exceedingly straightforward. The suggested technique can be adapted for the advancement of further enzyme-based biosensors [34].

Barman et al. created an electrochemical L-glutamic acid biosensor utilizing carboxyl-terminated (Crbxl)-RGO and PtNP [35], building upon Hummer’s work [36]. They initially synthesized Crbxl-RGO and anchored the composite material in the sensing area. The stability of the immobilized glutamate oxidase was subsequently improved through treatment with 1-ethyl-3-(3-dimethylaminopropyl)-carbodiimide and activation of the –COOH groups [37]. Crbxl-RGO was drop-cast onto the Au substrate, and PtNPs were electrodeposited on the Crbxl-RGO/Au modified substrate. The sensor displays a limit of detection (LOD) of 0.1 μM, a sensitivity of 973 ± 4 μA Mm^−1^ cm^−2^, and a linear range of 0.004–0.9 mM, demonstrating exceptional specificity and selectivity [35].

Nguyen et al. developed a printable glutamate biosensor employing PtNPs and a conductive polymer composite by the direct ink writing technique, that exhibit enhanced functionality compared to earlier models, achieving a sensitivity of 5.73 ± 0.078 nA μM^−1^ mm^−2^, a LOD of 0.03 μM, a reaction time of one second or less, and a linear range extending from 1 μM to 925 μM. Furthermore, the sensor’s sensitivity may be significantly enhanced, enabling its application for *in vivo* detection. The analysis revealed that successive evaluations of the device exhibited around a 3% variation in sensitivity between the initial and eighth trials. Glutamate sensors based on metal oxides have effectively identified glutamate in the absence of enzymes; however, enzyme-free glutamate sensors utilizing PtNP remain in the developmental phase. Glutamate sensors utilizing PtNP for *in vivo* applications and the food sector remain in the developmental stage. Therefore, going forward, the main goal of research and development should be to create new enzyme-free sensors for Pt-based nanomaterials that are very selective, very sensitive, mobile, able to detect *in vivo*, and economically viable [38].

A summary of the analytical parameters for the biosensors used for the detection of glutamate is presented in Table 1.

**Table 1:** Comparison of the analytical performance of different electrodes for glutamate detection.

Electrode	Linear Range	Detection Limit	Sensitivity	Response Time (s)	Refs.
GR/Co_3_O_4_/CS	4.0 × 10^−6^–6.0 × 10^−4^ M	2.0 × 10^−6^ M	7.37 µA mM^−1^ cm^−2^	25	[34]
Au/Crbxl-RGO/PtNPs	0.004–0.9 mM	0.1 μM	973 ± 4 μA mM^−1^ cm^−2^	-	[35]
Pt-C-PEDOT:PSS	1–925 μM	0.03 μM	5.73 ± 0.078 nA μM^−1^ mm^−2^	≤1	[38]

#### 2.1.2. Glucose Detection

The advancement of biosensors and the enhancement of parameters such as response time, sensitivity, and detection limit have led to the creation of crystalline porous materials featuring a periodic network structure composed of organic linkers, metal ions, and MOF [39]. They possess changeable pore dimensions, exceptional porosity, and substantial hydrophilic/hydrophobic groups [40]. The MOF-74 group is regarded as a robust framework material with commendable temperature and moisture stability [41].

Carbon-based 3D hybrid materials have emerged as a possible candidate for enhanced efficiency in biosensor applications. The study of Uzak et al. concentrated on integrating PtNP coated on reduced graphene oxide (RGO) with zinc metal–organic framework (Zn-MOF-74) and glucose oxidase (GOx) for the fabrication of a biosensor. The electrochemical analysis of the biosensor demonstrated that the constructed electrode is appropriate for application in an electrochemical glucose biosensor, exhibiting an extensive and rapid response time, linear range, elevated sensitivity, and minimal detection threshold. RGO-PtNP improved the conductivity of the nanomaterial. Furthermore, RGO and Zn-MOF-74 augmented the porosity and surface area, facilitating improved enzyme immobilization and deposition of PtNP. The biosensor’s repeatability and reproducibility were assessed in phosphate-buffered saline (PBS) (0.1 M, pH 7.4) with 6 mM glucose. The relative standard deviation (RSD) of the current response from 10 consecutive measurements with the same biosensor was 7.1%, signifying satisfactory repeatability. The RSD of the current response for six separately constructed biosensors was 6.6%, indicating strong repeatability of the fabricated biosensor. The findings indicate that the commendable repeatability, reproducibility, and stability of the nanomaterial imply its prospective applications as a sophisticated material for the production of commercial biosensors. Enzyme immobilization on MOF decreased enzyme aggregation and limited structural alterations of GOx, hence improving stability, reusability, and enzymatic activity. Furthermore, the engineered biosensor exhibited commendable accuracy in glucose measurement within cherry juice [42].

Savk et al. produced Pt–Ni nanocomposites loaded with activated carbon (PtNi/AC). In order to create an electrochemical glucose biosensor that does not need enzymes, the improvement on the GCE surface was made using PtNi/AC. The PtNi/AC can efficiently catalyze the oxidation of glucose even without an enzyme. The addition of glucose significantly modifies the strength of the current. The current density gradually increased, reaching a stable current within 2 s. The sensitivity reaches 40.9 mA mM^−1^ cm^−2^, the linear range extends from 0.025 to 12 mM, and the LOD reached 0.052 μM [43].

In the future, the development of glucose biosensors should focus on creating regenerative Pt-based biosensors or integrating them with alternative materials such as molecularly imprinted polymers, ligands, peptide networks, and adhesions. This guideline indicates the need for a more uniform assessment of the analytical efficacy of glucose biosensors to provide accurate and reliable assays.

Table 2 provides an overview of the analytical parameters utilized by glucose detection biosensors.

**Table 2:** Comparison of the analytical performance of different electrodes for glucose detection.

Electrode	Linear Range	Detection Limit	Sensitivity	Response Time (s)	Refs.
GCE/Zn-MOF-74-rGO-PtNPs-GOx	0.006–6 mM	1.8 μM	64.51 μA mM^−1^ cm^−2^	40	[42]
GCE/PtNi@AC	0.025–12 mM	0.052 μM	40.9 mA mM^−1^ cm^−2^	2	[43]

#### 2.1.3. Urea Detection

Excessive serum urea buildup can lead to shock, dehydration, and renal disorders. Consequently, precise measurement of urea is essential in the therapeutic of many problems. Biosensors provide a swift option for urea detection due to their elevated sensitivity, specificity, and durability.

The nickel oxide (NiO) nanoparticles also offer the potential to interact with urease (Urs), establish an electrochemical response in the biosensor, and detect urea. To achieve immobilization, it is sufficient to obtain a composite with a polymer, such as polypyrrole (PPy), and deposit it over a conductive electrode, such as Pt, to enhance the response of the device [44].

Therefore, Hosseinian et al. obtained a Pt/PPy–NiO electrode for urea detection. Urease was immobilized on the surface of Pt/PPy-NiO by physical adsorption. The Pt/PPy-NiO/Urs exhibited a suitable linear response range of 0.7–26.7 mM (R^2^ = 0.993), with a high sensitivity of 0.153 mAmM^−1^cm^−2^, and adequate selectivity, and long stability (10 weeks) [44]. The study by Hosseinian et al. progressed with the creation of an amperometric urea biosensor utilizing immobilized Urs on PPy and a macroporous PPy (MPPy)-modified Pt electrode. Glutaraldehyde (GA), a cross-linking agent, was employed to enhance the efficiency and stability of the enzyme immobilized on the electrode surface. The altered biosensor incorporating MPPy exhibited significant stability and optimal selectivity for urea in physiological specimens. The biosensor was tweaked multiple times under identical settings to assess its repeatability. The modified electrodes were evaluated in a 5 mM urea solution, and the results, with a RSD of 4.3%, indicated that the biosensor exhibited satisfactory repeatability [45].

The exploitation of the many properties of PtNP is hindered by the intricacies of the electrode process and the constraints of detection time; therefore, developing a urea biosensor based on Pt must adhere to specific protocols. The development should implement straightforward techniques to enhance anti-interference efficacy. Additional research should focus on the analysis of blood samples. Compound NPs should be used to improve the activity of Urs. Ultimately, it must implement straightforward and efficient strategies to improve the sensor’s stability and fulfill commercial requirements [45].

Depending on the number of metals involved, metallic nanoparticle-based biosensors can be found in many combinations, including mono-, bi-, tri-, and quadrometallic NPs [46]. A single metal makes up monometallic NPs, which should provide plasmonic properties for the system while guaranteeing a biomedical use based on excellent biocompatibility. These materials can guarantee a variety of effects and are based on a singular type, such as Ag, Au, Ti, Al, Zn, Bi, or Si [47]. Although the majority of research focuses on antibacterial action, their incorporation into optical or electronic equipment can guarantee accurate fluid detection [46]. In addition to their effects as antimicrobial and antioxidant agents [48], metal oxides like Ag_2_O, ZnO, TiO_2_, CuO, MgO, Al_2_O_3_, and Fe_3_O_4_ have proven useful in a variety of biological contexts, include medication delivery methods [49], biological sensors [50], specific treatments using imaging techniques in medicine [51], the food sector [52], optically and digital equipment [53]. Applications for metallic and metal oxide particles include biosensors for agriculture, medicine, and the environment [46].

Bimetallic nanocomposites incorporate metal particles within a ceramic or polymeric matrix, contingent upon the intended purpose. These composites are multifunctional, typically integrating the plasmonic response of NPs with the mechanical or elastic capabilities of the encompassing matrix. When encapsulated within a shell or incorporated into thin films, NPs can function effectively while mitigating the hazards associated with the use of biosensors. The catalytic, electrical, and optical capabilities are enhanced while preserving the nanoscale dimensions, and in addition, the polymeric matrix offers the patient both support and comfortable conditions [54].

Multimetallic NPs, referred to as tri- or quadrometallic particles, have enhanced and adjustable optical, electrical, and catalytic capabilities relative to mono- and bimetallic particles. The coexistence of several metals within a single material result in a synergistic impact due to the amalgamation of electrical reactivity, diverse morphologies, surfaces, or chemical structures. In biosensors, these metal element connections enhance the device’s activity, enabling specific applications.

Table 3 presents a summary of the analytical parameters for the biosensors used in urea detection.

**Table 3:** Analytical performance comparison of several electrodes for the detection of urea.

Electrode	Linear Range	Detection Limit	Sensitivity	Response Times (s)	Refs.
Pt/PPy-NiO/Urs	0.7–26.7 mM	0.0016 mM	0.153 mA mM^−1^ cm^−2^	5	[44]
Pt/MPPy/GA-Urs	0.5–10.82 mM	0.208 mM	0.0432 mA mM^−1^	5	[45]

### 2.2. Polymer Nanocomposites Used in Biosensors

Currently, polymer nanocomposites are the most favored nanomaterials [55]. In polymeric nanocomposites, a filler, either organic or inorganic, is incorporated into a polymer matrix at a minimal volume proportion.

Polymer nanocomposites have garnered considerable attention from academics in the healthcare industry due to their substantial potential to enhance engineering applications. The characteristics of polymer nanocomposites are determined by the nature of the nanomaterials incorporated into the polymer matrix, encompassing their concentration, dimensions, form, and interactions with the polymer matrix [56]. Polymer nanocomposites have been employed in several applications, including wastewater treatment [57,58], tissue engineering [59], electrochemical sensors [60,61], drug delivery [62,63], food processing [64], transparent thin films [65,66], and biomedical applications [67,68].

Polymeric and biopolymeric nanocomposites denote a hybrid structure whereby a polymer matrix serves as a substrate, while nano-sized organic or inorganic constituents function as fillers. Commonly, the polymers poly(lactic acid), poly(ethylene oxide), poly(lactic-co-glycolide), poly(N-isopropyl acrylamide), and in the construction of polymeric nanocomposites, polyurethanes have been used as the matrix phase. Biopolymeric nanocomposites, commonly referred to as “bio-nanocomposites,” “bio-hybrids,” and “green nanocomposites,” consist of nanosized additives integrated within naturally occurring polymers such as cellulose, chitin, collagen, silk, keratin, alginate, lignin, starch, and polyhydroxyalkanoates. The incorporation of nanoscale filler materials into the polymeric matrix yields enhanced mechanical, thermal, and optical properties. Filler materials function as molecular connectors, improving and regulating dimensional stability, flexibility, strength, toughness, durability, thermal stability, conductivity, optical characteristics (color and transparency), dimension, distribution, and form [69]. Nanofillers utilized in the preparation of polymer nanocomposites include organic materials such as CNTs and GR, as well as inorganic elements like silicates and metal/metal oxides [70,71]. Choosing the right filler and matrix may affect the characteristics of polymeric nanocomposites. Filler choice has a dramatic effect on structural and functional characteristics, while the type of polymer matrix greatly affects hydrophobic properties, transparency, durability, regulate ionizability, crystallinity, functioning, biological compatibility, and bio-degradability. Therefore, various mixtures of nanofillers may be synthesized to create unique polymer nanocomposites. By selecting filler nano-objects with the precise properties applicable across various domains, including medicine, diagnostics, food wrapping, optical electronics, biological sensing, bioimaging, the engineering of tissues, personal care products, energy, this variety enables application-focused strategies. Extensive research on polymeric nanocomposites has significantly enhanced sensor performance, facilitating the development of numerous innovative biosensors in recent years [72,73]. Quantum dots (QDs)–polymeric nanocomposites demonstrate superior fluorescence qualities suitable for optical biosensors, whereas CNTs–polymeric nanocomposites significantly boost mechanical properties applicable to optoelectronic sensing devices [3]. The application of polymeric nanocomposites facilitates tailored designs; for instance, quantum dot–polymeric nanocomposites demonstrate superior fluorescence characteristics suitable for optical biosensors, whereas carbon nanotube–polymeric nanocomposites significantly improve mechanical properties, making them applicable in optoelectronic sensing devices. It offers further critical performance metrics, such as enhanced sensitivity and selectivity, reduced detection thresholds, excellent repeatability, and stability, achieved by a substantial and readily modifiable surface area, increased electrical conductivity, and rapid electron transfer rates [74].

**Figure 4:**
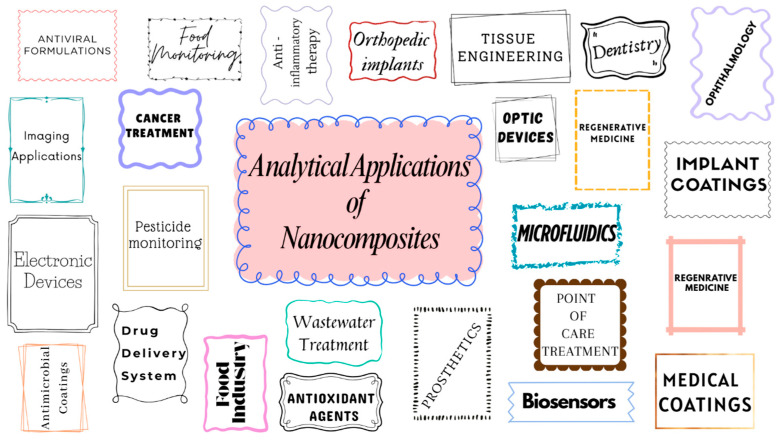
Potential analytical applications of nanocomposites.

Silver nanoparticle–polymer nanocomposites (AgNP–PNCs) signify an important progression in biological material science, combining the strong antibacterial characteristics of AgNPs with the structural adaptability of polymer matrices. The interplay between these nanocomposites makes it possible to increase infection control, mechanical stability, and regulated medication administration. As a result, these nanocomposites are especially suitable for applications such as wound healing, medical coatings, tissue engineering, or biosensors. Enhanced control over particle shape, dispersion, and stability has been achieved as a result of recent advancements in synthesis and functionalization. This has led to an improvement in the clinical and translational uses of AgNP–PNCs. Despite this, there are still concerns about cytotoxicity, long-term stability, immunological answer, and scalability. These concerns need the implementation of advancements in functionalization of surfaces, crossover methods, and biocompatibility evaluations systematically. The choice of constituents for AgNP–PNCs is crucial as it influences their biocompatibility, mechanical characteristics, antibacterial effectiveness, and the reliability of their functions in biological contexts. Silver nanoparticles can be effectively incorporated into a variety of matrices, including polymers that are synthetic or natural, inorganic components, and materials made of carbon. This allows for the development of advanced nanocomposites that have properties that can be tailored to specific applications, such as wound dressings, implant coatings, biosensors, and drug delivery systems, as summarized in Figure 4.

#### 2.2.1. Natural Polymers

Natural polymers are extensively used in the production of AgNP–PNC due to the fact that they can interact with living systems, are biocompatible, and biodegrade. Chitosan, a polysaccharide obtained from chitin, demonstrates antibacterial and anti-inflammatory characteristics, rendering it suitable for wound healing and medication delivery purposes [75]. As a result of its capacity to enhance the mechanical stability of nanocomposites, gelatin, which is derived from collagen, is commonly utilized in medical and tissue engineering applications. In the field of regenerative medicine, biomaterial production is conducted [76]. As a result of its ability to generate hydrogels when calcium ions are present, alginate, which is an anionic polymer that occurs naturally, is used extensively in the production of wound care products and tissue scaffolds [77]. Cellulose-based materials, which are known for their durability and biodegradability, serve as a stabilizing matrix in applications that make use of AgNPs–PNCs. These materials are suitable for coatings, films, and biological applications [78]. Starch-based AgNPs–PNCs have shown encouraging outcomes as biodegradable drug delivery carriers, offering improved stability and controlled release characteristics [79]. The unique self-assembling characteristics and exceptional biocompatibility of silk fibroin, a byproduct of silkworms, are drawing increasing interest in its potential use in medical regeneration and engineering of tissues [80]. Because of its importance in both water retention and wound healing, hyaluronic acid is a great ingredient for hydrogels and injectable medication carriers [81]. A crucial structure protein, collagen type is widely used in medical regeneration and tissue structuring [82]. Biomedical coatings, antiviral treatments, and therapy for inflammation make use of carrageenan, a sulfated polysaccharide produced from red seaweed [83].

#### 2.2.2. Synthetic Polymers

A wide variety of synthetic polymers are used in AgNPs–PNCs for biomedical applications. These include PDMS, PEG, PVA, PLGA, PEO, PLA, PU, PMMA, and PCL, which are known for their flexibility, stability, and compatibility with medical applications. PDMS exhibits exceptional flexibility and biocompatibility, making it suitable for applications in microfluidics, tissue engineering, and implantable devices [84]. PEG’s controlled release properties [85] and ability to increase drug solubility make it an important component of hydrogels for targeted imaging. It also has outstanding mechanical properties and biocompatibility, thereby being appropriate for use as wound dressings and medicinal coatings [86]. Biodegradable PLGA is frequently utilized in medication delivery and tissue engineering, whilst PEO’s solubility in water facilitates analogous applications [87]. The exceptional flexibility and mechanical stress endurance of polyurethane makes it a popular material for implanted medical devices and prostheses [88]. PMMA, a widely recognized substance in ophthalmology and dentistry, has exceptional tissue compatibility. Its structural integrity, rendering it a favored option for intraocular lenses, bone cement, and dental implants [89].

#### 2.2.3. Inorganic and Carbon-Based Materials

In AgNPs–PNCs, structural strengthening made of inorganic materials, which improves the mechanical properties, stabilization, and biological function of the material. As a result of their amazing resilience and high surface area, silica NPs are well suited for use in scanning applications, biological sensors, and specific drug delivery [90]. Titanium dioxide (TiO_2_), esteemed for its biocompatibility and photocatalytic attributes, is integrated into AgNPs–PNCs for antimicrobial coatings, dental applications, and orthopedic implants [91]. Because of its bioactivity and structural similarity to genuine bone, hydroxyapatite (HA) is a crucial component of bone substitutes, transplants, and tissue engineering [92]. Wound treatments, biological sensors, and coatings that are antimicrobial can benefit from zinc oxide’s (ZnO) antibacterial, blocking of ultraviolet radiation, and piezo-electric properties [93]. For applications in the treatment of cancer, medical imaging, and antimicrobials, AgNP-PNCs with AuNPs added improve their plasmonic and photothermal properties [94].

The role of carbon materials is crucial in both the modifiers used in electrochemical sensors and the materials for working electrodes. A variety of porous carbon materials, such as carbon quantum dots (CQDs), RGO, graphene oxide, and CNTs, have been effectively utilized in the development of sensors. The materials offer active sites and an extensive surface region for adsorption, thereby improving the ability of analyte detection. Pure carbon materials can effectively provide both selectivity and sensitivity for detection applications. Furthermore, their ability to form robust covalent bonds with various substances has resulted in the synthesis of a wide range of nanomaterials or composites for electrochemical sensing applications. Frequently, these materials act as foundations for supplementary modifier-substances, including metal-oxides, metals, and conductive polymers.

Carbon-based materials, including GR, CNTs, and carbon dots, have garnered considerable attention in the synthesis of AgNP–PNC because of the remarkable antimicrobial, electrically conductive, and mechanically strong qualities they possess. When it comes to biosensors and drug delivery systems, GR’s large surface area makes it ideal for immobilizing AgNPs, which improves their conductivity and stability [95]. Carbon nanotubes, characterized by their elevated aspect ratio and distinctive electrical characteristics, are under investigation in order to achieve specific medication delivery and antimicrobial coatings. Bioimaging and diagnosis of cancer are two areas where carbon dots have shown promise because of their luminous properties and low cytotoxicity.

##### Carbon Nanotubes in Biosensors

Characterized by exceptional mechanical, electrical, electrocatalytic, and thermal capabilities, carbon in its allotropic form is referred to as CNT. The number of constructed walls determines its characteristics, which may be described as a rolled-up GR sheet [96]. CNTs have a lot of promise as biosensors due to their unique shape. Diameters of multi-walled carbon nanotubes (MWCNTs) may reach 100 nm, in contrast to the lowest diameters of single-walled carbon nanotubes (SWCNTs), which range from 1 nm to 2 nm. Various flaws are bound to be present in them because of the various layers surrounding them. In contrast, double-walled carbon nanotubes (DWCNT) have many of the characteristics of SWCNT and fewer flaws than MWCNT, placing them in the middle of the two extremes [97].

The benefits GOx, AgNPs, MWCNTs, and tropine-amino acid-based ionic liquids (TABILs) were combined by Peng et al. [98] to produce a novel glucose biosensor with potential uses in the food industry. A series of modified electrodes was developed and tested after a methodical discovery of the impact of different ionic liquids on GOx and their intermolecular interactions. After careful study, it was found that the TABIL made from butyl-substituted tropinol and serine was the best choice. The electrode constructed with [BuTr][Ser]/Ag/MWCNTs had the greatest active area, 1.68 times greater than the bare electrode. At a working potential of −0.45 V and 25 °C, the electrode was adjusted to detect glucose in PBS (pH = 7) after further adjustment with 5 mg mL^−1^ of GOx. The electron transfer rate was 0.91 s^−1^, the Michaelis-Menten constant was 5.10 mM, and the GOx coverage was 2.16 × 10^−9^ mol cm^2^. When it came to the identification of glucose in possible food items, the results of the tests were satisfactory for genuine honey samples, which indicated that it had a good performance.

**Scheme 1:**
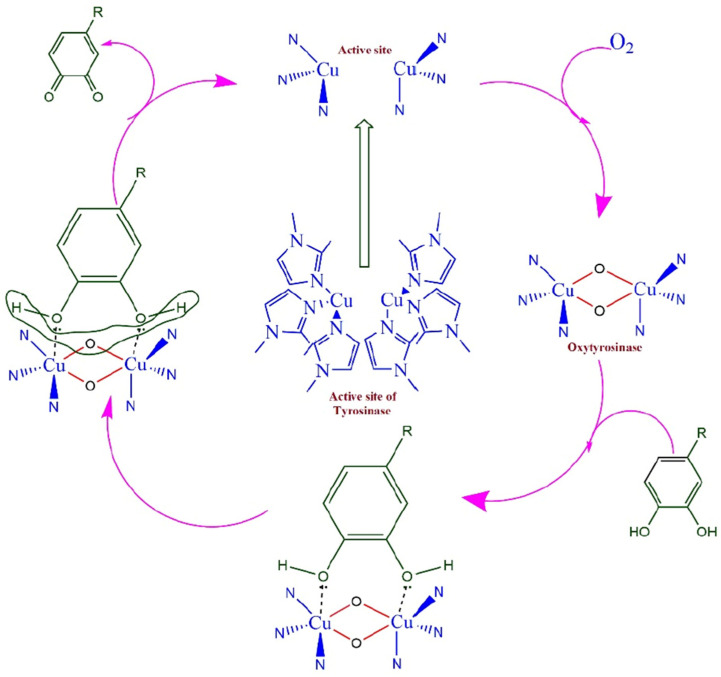
The mechanism of the tyrosinase catalytic process is shown in a schematic diagram. Reproduced [99] with the permission of the Creative Commons Attribution 4.0 International License (http://creativecommons.org/licenses/by/4.0/ (accessed on 24 October 2025)). Copyright 2025 by CEE.

The polyphenolic flavonoid known as rutin (RU) is an organic compound that has high antioxidant effects and may be found in a wide variety of plant-based sources from nature. As a result of the natural antioxidant action that it has, RU prevents hazardous cancer cells from forming and strengthens the blood vessels. The purpose of the work exhibited by Gopal et al. [99] is to construct a highly sensitive electrochemical biosensor for the detection of RU. This will be accomplished by altering a GCE with MWCNTs and dropping the tyrosinase enzyme (Ty) onto the surface (Scheme 1). The techniques of CV, electrochemical impedance spectroscopy (EIS), and Tafel plot investigations were used to highlight the assessment of the performance of the biosensor. The electrochemical redox behavior of RU was exhaustively explored, and a potential redox mechanism was hypothesized as a result of this investigation. In the course of the investigation into the impact that the pH of the supporting electrolyte PBS has on the electrochemical behavior of RU, it was determined that a pH of 6.0 is the most suitable for future research. Upon conducting the kinetic investigations, it was discovered that the detection process was regulated by adsorption. The characteristics of the surface coverage concentration (Γ), the heterogeneous rate constant (k_s_), and the charge transfer coefficient (α) were determined by using the scan rate experiments. The biosensor had remarkable analytical performance, as shown by its LOD of 4.47 × 10^−8^ M and its limit of quantification of 1.55 × 10^−7^ M, as determined by the linear regression equation. The biosensor’s potential for reliable and robust detection of RU was shown by testing that demonstrated its repeatability, reproducibility, and stability. These tests also demonstrated that the biosensor has a practical use.

It is well recognized that serum homocysteine (Hcy) may serve as an accurate indicator of neurological, cardiovascular, and cerebrovascular disorders. Consequently, it is critical for diagnosis and prevention to have a way to test HCy that is sensitive, precise, and quick. Electrochemical techniques have been extensively used for the determination of specific biomolecules due to their simplicity, low cost, and rapid response. Using a portable mini-potentiostat as its foundation, the study conducted by Shi et al. [100] developed the HCy electrochemical biosensor that incorporates screen-printed electrodes modified with Au and manganese (IV) nanomaterials on MWCNTs (Au@MnO_2_/MWCNTs). The proposed electrochemical biosensor showed a wide-linear range (5–125 μM), high sensitivity (0.01110 μA μM^−1^), and low LOD (0.6173 μM) for HCy, with a detection of only 400 s, all thanks to the exceptional electrochemical activity and catalytic capability of Au@MnO_2_/MWCNTs. Serum HCy levels were accurately determined using it, and the findings corroborated those of an autonomous clinical chemistry analyzer. In addition, the simple and transportable electrochemical gadget presents a viable approach for in-home health monitoring and on-site study.

**Figure 5:**
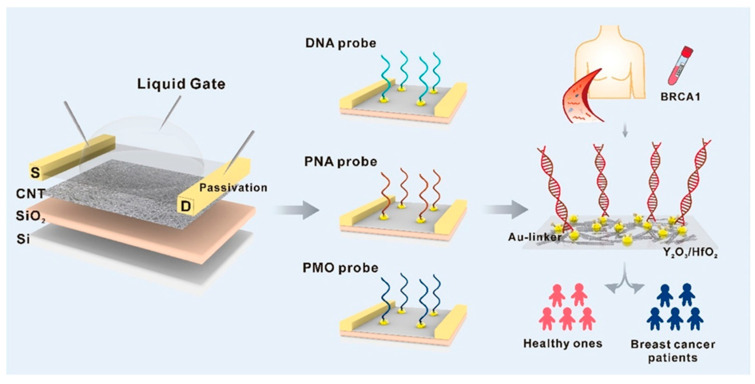
A schematic representation of the probe-screened FG CNT-FET is shown for the purpose of ultrasensitive BRCA1 detection. Reproduced [101] with the permission of the Creative Commons CC-BY-NC-ND. Copyright 2025 Elsevier B.V.

When it comes to liquid biopsy and early identification of breast cancer, BRCA1 gene testing is a savior since the conventional methods that are now in use, such as gene sequencing, are not only costly but also time-consuming and difficult. A field-effect transistor (FET) DNA sensor built from semiconductor CNTs with a floating gate (FG) structure and a high bilayer gate dielectric of yttrium oxide/hafnium oxide (Y_2_O_3_/HfO_2_) was built by Liu et al. [101]. To identify the optimal BRCA1 sensor design, the authors initially thoroughly examined the detection performance of three nucleic acid probes: DNA, phosphorodiamidate morpholino oligos (PMO), and peptide nucleic acid (PNA) (Figure 5). With a low LOD of 1.38 aM and high specificity, the PNA-functionalized FG CNT-FET sensor was distinguishable. And since the bilayer dielectric was passivated, the created sensor showed excellent anti-interference capacity in serum BRCA1 testing as well. The important thing is that this technology was able to discriminate between healthy people and breast cancer patients, which agrees with the gene sequencing findings. This study exemplifies an ideal analytical framework for efficient, accurate, and user-friendly liquid breast cancer biopsies.

An outline of the analytical criteria for biosensors that employ carbon nanotubes to detect biological analytes is summarized in Table 4.

**Table 4:** The analytical parameters for the carbon nanotube based biosensors used to detect biological analytes.

Electrode	Linear Range	Detection Limit	Sensitivity	Refs.
Ty/MWCNTs/GCE	-	4.47 × 10^−8^ M	-	[99]
Au@MnO_2_/MWCNTs/SPE	5–125 μmol L^−1^	0.6173 μmol L^−1^	0.01110 μA μM^−1^	[100]
FG CNT-FET PNA	-	1.38 aM	-	[101]

##### Quantum Dots-Based Biosensors

Nanoparticles having dimensions that are fewer than 10 nm are referred to as QSs. These NPs are also referred to as fluorescent semiconductor nanocrystals.

Recently, they have attracted a tremendous amount of interest in revolutionary biosensing technological infrastructure. Within the realm of sensing applications, QDs are employed extensively by researchers due to their outstanding optical, electrical, and size-dependent luminescent capabilities [102]. Furthermore, it has been shown that QDs possess outstanding charge carrier transport capabilities and a high surface-to-volume ratio, both of which have the potential to improve the value of a biosensor. Due to their remarkable qualities, which include low toxicity, greater soluble properties, chemically stable behavior, and adaptability, CQDs have been significant in investigations on frontline sensing [102,103]. In this regard, CQDs have become notable. For example, Wei’s group developed a biosensor that is based on CQDs for the purpose of detecting acrylamide (AM), a carcinogenic ingredient, from food goods in a very short amount of time, thanks to their efforts. The fluorescence of the CQDs was suppressed by combining them with ssDNA after they were synthesized using a hydrothermal process that only required one pot. Due to the establishment of a hydrogen bond between ssDNA and AM during the process of adding AM, very few free DNAs were left behind, which ultimately resulted in a reduction in the amount of fluorescence that was produced by CQDs. The fluorescent property of CQDs and the strong connection between ssDNA and AM were employed in the immediate identification of AM from bread crumbs, which resulted in a LOD of 2.41 × 10^−8^ M [103]. The identification of cysteine in solutions was accomplished by Kamaci’s group via the development of biosensors based on ZnO QDs. They devised an innovative method for the development of a fluorescent probe by making use of ZnO QDs that had been treated with melamine (MEL). The fluorescent biosensor that was based on ZnO QDs had a more robust fluorescence response when it attempted to detect cysteine. Its linear range was between 0.1 and 600 µM, and its LOD was 0.642 µM [104].

The development of biosensors that use graphene quantum dots (GQDs) as energy donors and GRNPs as energy receivers has made it feasible to identify tiny lung cell carcinoma at an early stage and in a quick manner. A significant LOD of 0.09 pg mL^−1^ was achieved by the fluorescent response research that was conducted for the purpose of detecting lung cancer. The response time was 17 min, and the detection range was expanded from 0.11 pg mL^−1^ to 1002 ng mL^−1^ [105]. Several researchers have created a wearable sensor that is based on textiles and is both simple and inexpensive. This sensor can detect glucose and hydrogen peroxide (H_2_O_2_) levels. Prussian blue was combined with CdSe QDs and RGO QDs by oil–water self-assembly engineering on a flexible ITO substrate to produce a nanofilm technology that is based on fabric composition. Specifically, the films demonstrated remarkable electro-chemical sensing capability, exhibiting a high sensitivity of 53.8 µA mM^−1^ cm^−2^ for H_2_O_2_ and 37.24 µA mM^−1^ cm^−2^ for glucose [106].

**Figure 6:**
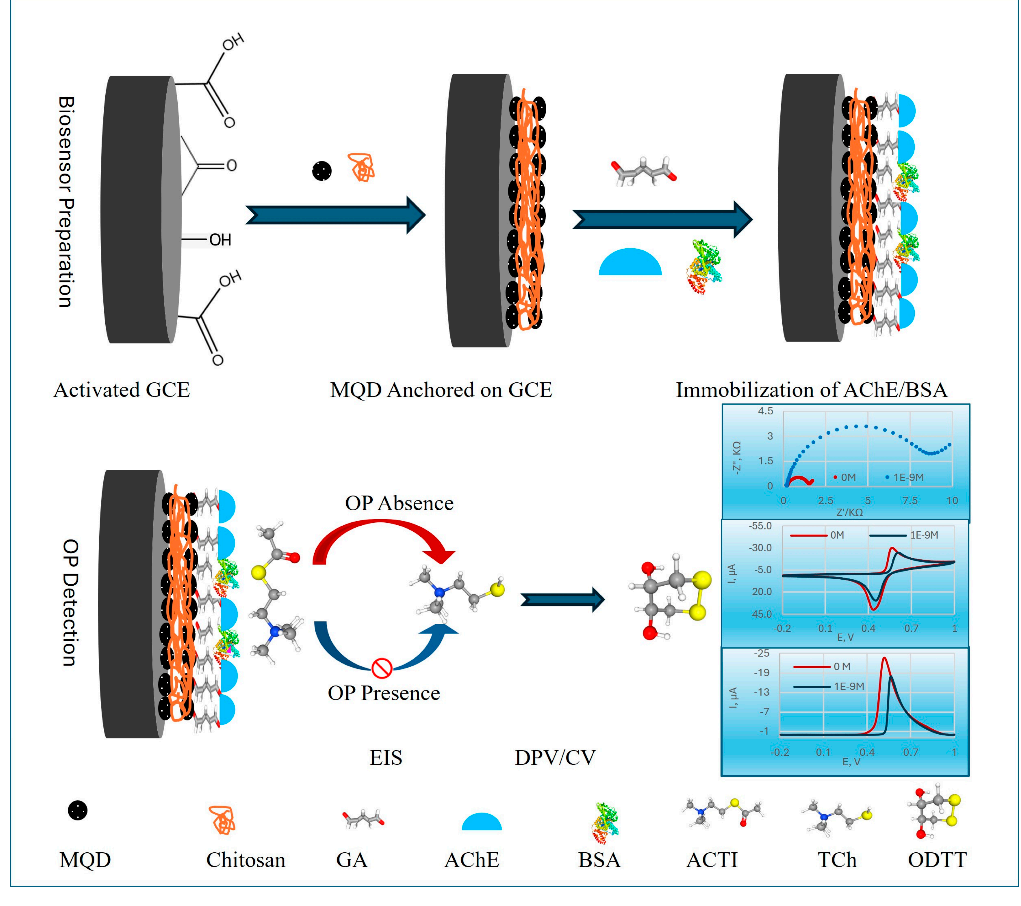
Schematic representation of the electrochemical OP biosensor based on MQDs. Reproduced [107] with the permission of the Creative Commons Attribution 4.0 International License (http://creativecommons.org/licenses/by/4.0/ (accessed on 21 October 2025)). Copyright 2025, MDPI.

Organophosphorus pesticides (OPs) are a serious threat to human health and the environment because they inhibit AChE. This is partly because they are used extensively and are hazardous. Because conventional techniques of detection are sometimes sluggish and expensive, there is an immediate and pressing need for technologies that are modern, sensitive, and easily obtained. Utilizing Ti_3_C_2_T_x_ MXene quantum dots (MQDs), which were synthesized by a hydrothermal process (Figure 6), in this work, proposed by Makani et al. [107], established a very sensitive electrochemical cholinesterase-inhibiting biosensor for OPs. The biosensor’s effectiveness was assessed using cyclic voltammetry, differential pulse voltammetry (DPV), and EIS. For chlorpyrifos, a model OP, the DPV approach was shown to be the most effective method, displaying a very low LOD of 1.0 × 10^−17^ M and a broad linear range of 10^−14^–10^−8^ M. Furthermore, the estimated inhibition constant for this methodology was 62 nM. When compared to in vitro cholinergic activity testing in bean beetle homogenates, the biosensor revealed a strong selectivity for OPs (chlorpyrifos, acephate, and glyphosate) over a non-target pyrethroid (permethrin). This was supported by different electrochemical signatures for each of the OPs. A high surface-to-volume ratio, quantum confinement effects, and greater conductivity of the MQDs are all responsible for the improved performance. Glutaraldehyde cross-linking and a CS matrix achieved robust enzyme immobilization, which further enhanced the performance. The findings offer a workable framework for the quick, sensitive, and targeted identification of OPs and demonstrate potential application value in environmental monitoring and public health defense.

The glioma is responsible for eighty percent of all primary brain tumors that are malignant and have a high death rate. Histopathological examination is the approach that is used to diagnose glioma; nevertheless, the invasive surgical procedures that are required for this test might result in cerebral edema or may impede neurological functioning. It has been shown that liquid biopsy is an effective technique for the identification of gliomas. The blood–brain barrier, on the other hand, limits the amount of circulating tumor cells (CTCs) that are present in the circulation. This presents a hurdle for the sensitive detection of glioma CTCs. This research aims to develop a sensitive technique for identifying glioma cerebral tumor cells, leveraging the unique qualities of nanocomposites and the specificity of Angiopep2 (Ang-2). Employing GQDs-nanoporous gold (NPG) nanocomposites used as the immobile platform and the Ang-2 protein as the biorecognition component, a new label-free impedimetric biosensor was successfully created by Wang et al. [108] for the detection of glioma cell tumor cells (CTCs). The GQDs were homogeneously built onto NPG, which resulted in the production of a new nanocomposite consisting of GQDs and NPG that has distinctive qualities in terms of both its structure and its function. In light of the fact that the GQDs-NPG nanocomposite had a high efficiency of electron transfer, the biosensor that was created demonstrated a wide detection range that extended from 1 to 1 × 10^6^ cell mL^−1^, while maintaining a minimum LOD of 1 cell mL^−1^. In addition, the glioma cell biosensor exhibited a powerful anti-interference capacity against a number of different cell lines, and the biosensor’s stability was maintained at 96% after being stored for a period of 21 days. Furthermore, the glioma cell biosensor identified the number of glioma cells in human blood samples, showing an exceptional level of consistency with the standard values supplied to the samples. As a result of this work, a new nanocomposite composed of GQDs and NPG was produced, and an electrochemical biosensor that was based on GQDs and NPG was first established for the detection of glioma CTCs. The biosensor made from glioma cells demonstrated a high level of sensitivity, a low detection limit, a powerful capacity to prevent interference, and a high level of stability in complicated biological structures. By successfully achieving the reliable detection of glioma cells in human blood, a good alternative has been provided for the identification of glioma CTCs using liquid biopsy and for the early diagnosis of glioma disorders.

Liu et al. created a zinc-doped molybdenum disulfide quantum dot (Zn-MoS_2_ QDs)-based dual-mode fluorometric and colorimetric biosensor for the Pax-5a gene by combining exonuclease-assisted recycling amplification with peroxidase-mimic DNAzyme [109]. Exonuclease III can release output DNA (oDNA) when the Pax-5a gene is present by cleaving the duplexes produced by the Pax-5a gene and hairpin DNA (HP). The G-rich DNA sandwich complex may be formed when magnetic beads (MBs) tagged with capture DNA (cDNA) hybridize with organic DNA (oDNA). The 1,3′-diaminobenzidine (DAB) oxidation by H_2_O_2_ was catalyzed by G-quadruplex/hemin peroxidase-mimicking DNAzyme, which was produced when the G-rich DNA remaining in the supernatant after magnetic separation bound hemin. The resulting brown oxidation product (oxDAB) was able to extinguish the fluorescence of Zn-MoS_2_ QDs at 406 nm and exhibited a clear absorption peak at 464 nm. This detection technology achieved exceptional sensitivity and specificity because of DNAzyme’s strong peroxidase activity, the recycling amplification method, and the magnetic separation technique. The limits of detection for the Pax-5a gene using colorimetric techniques were 1.12 pM, and fluorometric methods were 0.52 pM. In addition, the detection technique was effectively used to determine the Pax-5a gene in blood samples from humans, its encouraging for its potential use in biochemical research and healthcare diagnostics.

An essential biomarker for assessing human renal function, cardiovascular illnesses, and inflammatory statuses is urokinase (UK), an enzyme protein mostly extracted from the urine of healthy people. To achieve precise and sensitive identification of the UK, Wang et al. [110] established a new sandwich-type ratiometric electrochemiluminescence (ECL) biosensor. For this biosensor, titanium dioxide magnetic beads (P25) served as the cathode luminophore, while tris(4,4′—dicarboxylic acid—2,2′—bipyridyl) ruthenium dichloride (Ru(dcbpy)^32+^/ICPn) was housed in a terbium monophosphate-guanosine infinite coordination polymer network (Tb—GMP ICPn). A favorable linear correlation was observed between the variations in ECL intensities and the concentration of UK within the range of 10^−2^ to 10^4^ ng mL^−1^, when potassium persulfate and tri-n-propylamine were used separately as co-reactants under optimized experimental conditions. The assays yielded LODs of 7.2 and 5.8 pg mL^−1^, respectively. In addition, this biosensor worked as expected when used to detect UK in real biological samples. Based on these results, the ratiometric ECL biosensor that was created has great potential for use in clinical detection and diagnosis.

**Scheme 2:**
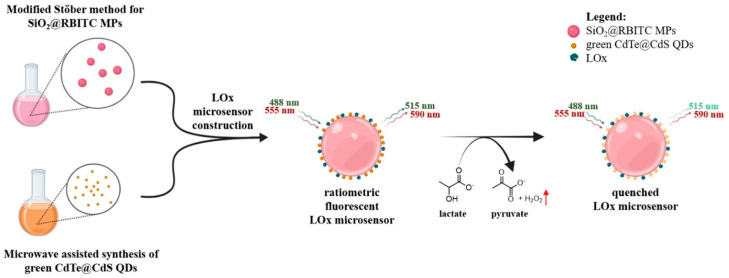
A schematic representation depicting the sequential fabrication of a ratiometric H_2_O_2_ sensor for lactate measurement using green-emitting CdTe@CdS QDs. Lactate oxidase catalyzes the conversion of lactate into pyruvate upon its introduction, producing H_2_O_2_ (red arrow), which proportionately suppresses the fluorescence of the QDs while maintaining a steady reference emission. Reproduced [111] with the permission of the Creative Commons CC-BY-NC-ND. Copyright 2025 Elsevier B.V.

As an important marker of anaerobic respiration and metabolic stress, lactic acid plays a significant part within a range of biological functions, notably in the metabolism of cells and the activity of muscles. A fluorescence quenching process is used by Grasso et al. [111] in their research, which results in the presentation of a ratiometric fluorescent biosensor in order to identify lactic acid. In addition to a reference probe that is covalently attached to silica microparticles (SiO_2_ MPs), which serves as the substrate, the sensor comprises a H_2_O_2_-sensing unit that is based on photoluminescent core–shell cadmium telluride@cadmium sulfide quantum dots (CdTe@CdS QDs). To catalyze the aerobic oxidation of l-lactate into pyruvate, lactate oxidase (LOx) is immobilized on the surface of the microparticles (Scheme 2). This process results in the production of H_2_O_2_. A proportionate dampening of the photoluminescence of the CdTe@CdS QDs is caused by H_2_O_2_ when the concentration of lactate increases from 0 to 30 mM. On the other hand, the reference fluorescence emission remains unchanged. To conduct a quantitative assessment of the sensor’s ratiometric response and colorimetric changes via the use of image analysis, confocal laser scanning microscopy (CLSM) was used to establish a lactate calibration curve. Using either rhodamine isothiocyanate (RBITC) or 7-(diethylamino)coumarin-3-carboxylic acid (7ACC1) as reference dyes, the researchers engineered two different variations in the microsensor: one with green-emitting CdTe@CdS QDs and the other with red-emitting CdTe@CdS QDs. Both of these dyes were co-immobilized with LOx enzyme on the surface of the microparticles. This was performed in order to demonstrate the versatility of this approach. Utilizing the H_2_O_2_-sensing capabilities of CdTe@CdS QDs in conjunction with the LOx enzyme activity, these cutting-edge microsensors provide a straightforward and efficient instrument for the quantitative measurements of lactic acid.

It is well established that T-cell lymphoma and acquired immune deficiency syndrome are both caused by the human T-lymphotropic virus type II (HTLV-II). Therefore, accurate detection of HTLV-II is essential for efficient therapy and early identification of the condition. Through the use of hybridization-initiated CdS QDs co-sensitized signal amplification, Chen et al. [112] were able to build a self-powered biosensor that was based on a photocatalytic fuel cell (PFC) for the detection of HTLV-II DNA. In order to drive the sensing process, the PFC was made up of a GR composite MoS_2_ (GR-MoS_2_) photoanode and a Pt cathode. This combination was able to provide a sufficient power output under visible light. Two probes that were partly complementary to the target DNA (tDNA) were able to efficiently detect and capture the target; this was accomplished by employing a capture probe DNA and a CdS QDs tagged report probe DNA. This resulted in a high level of selectivity. The co-sensitization action between CdS QDs and the photoanode material GR-MoS2 efficiently enhanced the electrical output signal of the PFC. By establishing a positive connection between the output power and the concentration of tDNA, it was possible to accomplish a signal-on DNA analysis assay that is capable of ultrasensitive detection down to 27 fM. Through the successful use of the developed sensor in the measurement of tDNA in human serum samples, the sensor demonstrated the highest possible level of accuracy and precision.

As a tumor marker for human epidermal growth factor receptor-2 (HER2), assessing its status is crucial in the examining, diagnosis, and observing of breast cancer. A groundbreaking method in cancer detection, Javazm et al. [113] research study detects HER^2+^ tumor cells in circulation by use of an electrochemical biosensor. The researchers examined the NPs’ electrochemical activity, size, shape, and morphology. Carbon quantum dot (CQD) nanoparticles, nickel-based MOFs, and polyethyleneimine (PEI) are placed onto the surface of graphite electrodes to form a composite. The platform enhances surface shape and characteristics, electrochemical activity, and biosensing efficiency after complete characterization. The graphenized graphite electrode-based biosensor has many remarkable features for detecting HER2-overexpressing SK-BR-3 cancer cells, such as a linear dynamic range of 100–500 cells mL^−1^ and an analytical LOD of less than 1 cell mL^−1^. High specificity, low cost, wide dynamic range, and repeatability are only a few advantages of this effective platform. All of these features point to the nanobiosensor’s promising future in clinical diagnosis and patient monitoring of HER2 levels in breast cancer.

An overview of the analytical parameters for the quantum dots based biosensors that are utilized for the detection of biological analytes can be found in Table 5.

**Table 5:** Parameters for the quantum dots based biosensors utilized in the detection of biological analytes.

Electrode	Linear Range	Detection Limit	Sensitivity	Refs.
Fluorescent biosensor based on CQDs and ssDNA	1.00 × 10^−7^–5.00 × 10^−3^ M	2.41 × 10^−8^ M	-	[103]
ZnO QDs, MEL	0.1–600 µM	0.642 µM	-	[104]
NSE/anti-NSE/amine-N-GQDs@AuNPs	0.1 pg mL^−1^–1000 ng mL^−1^	0.09 pg mL^−1^	-	[105]
PB-RGO-QD	0–5 mM	1.73 µM	31.6 µA mM^−1^ cm^−2^	[106]
AChE/CS/MQD	1.00 × 10^−14^–1.00 × 10^−8^ M	1.0 × 10^−17^ M	-	[107]
Ang-2/GQDs-NPG/GCE	1–1 × 10^6^ cell mL^−1^	0.139 cell mL^−1^	2005.58 ΩmL cells^−1^	[108]
Ru(dcbpy)32 + @Tb-GMP/GCE	1.00 × 10^−2^–1.00 × 10^4^ ng mL^−1^	5.8 pg mL^−1^	-	[110]
Label-free Ab@PEI@CQDs@Ni-MOF nanocomposite	100–500 cells mL^−1^	1 cell mL^−1^	-	[113]

### 2.3. Ceramic Matrix Composites Used in Biosensors

Ceramic matrix composites (CMCs) have become increasingly favored due to their enhanced properties compared to traditional ceramics. The properties of CMCs are influenced by multiple elements, such as the volume and distribution of the resulting phase, along with the method of synthesis employed. A range of CMCs has been developed using various synthesis methods tailored to specific application areas (Table 6).

Ceramic matrix composites, comprising inorganic ceramics primarily consisting of metal oxides and incorporating additional metal oxides such as CuO, ZnO, Fe_3_O_4_, TiO_2_, and SiO_2_, along with carbon materials, polymers, or other nanomaterial particles, are extensively utilized in catalysis, energy storage, and electrochemical sensing. This material has exceptional advantages, such as superior chemical stability, effective adsorption capabilities, tunable electrical properties, and robust material compatibility, thereby significantly advancing the electrochemical detection of cardiac troponin I (cTnI) [114]. Sriram Muthukumar et al. created flexible disposable electrochemical biosensors utilizing vertically oriented ZnO nanostructures for the quick simultaneous detection of cTnI and cardiac troponin T (cTnT). Electrodes were fabricated by cultivating ZnO nanostructures on working electrodes, utilizing these nanostructures to restrict target molecules for the simultaneous identification of cTnI and cTnT in human serum, achieving a limit of 1 pg mL^−1^ [115]. This platform is the inaugural one to accomplish simultaneous multiplex detection through various configurable approaches. The affinity sensing approach is expected to be utilized for the identification of other biomarkers with therapeutic translational potential. In the realm of nanomaterial and semiconductor device integration, Fathil et al. identified device architecture innovation as a pivotal advancement, attaining significant enhancements in detection sensitivity and signal transduction efficiency via the amalgamation of FET technology and ZnO nanofilms. Zinc oxide nanoparticle (ZnO-NP) thin film materials were deposited on the channel using sol–gel and spin-coating processes. The sensitivity of the method was much increased up to 9%·(g/mL)^−1^, and a LOD for cTnI down to 1.6 fg mL^−1^. This paved the way for the future development of innovative FET biosensors utilizing cutting-edge nanomaterials [116]. Khushaim et al. successfully utilized FET biosensing technology, employing indium gallium zinc oxide (IGZO) as a superior semiconductor channel in conjunction with nanosheet materials, to attain a detection range of 0.01–1000 ng mL^−1^, in the context of advancing biosensing technology for integration and device-oriented applications. The LOD was 0.0066 ng mL^−1^ [117]. This sensing approach employs AuNPs-modified porous carbon nitride (PCN) as an intermediary between solid-state devices and cTnI, characterized by its porous structure and extensive surface area, facilitating aptamer attachment. This offers a robust reference for improving the integration of cTnI and transistors to identify other biomolecules.

**Table 6:** Frequently used biosensors based on quantum dots for the detection of biological markers.

Electrode	Linear Range	Detection Limit	Sensitivity	Refs.
A flexible, disposable biosensor comprising vertically oriented zinc oxide (ZnO) nanostructures	-	1 pg mL^−1^	-	[115]
ZnO thin film with chemical linkers and bio-receptor	-	1.6 fg mL^−1^	-	[116]
IGZO/PCN-Au NPs	0.01–1000 ng mL^−1^	0066 ng mL^−1^	0.6 µA (ng/mL)^−1^	[117]

## 3. Gold Nanoparticles Used in Biosensors

### 3.1. Gold Nanoparticles in Biosensors

Due to their structure, dimensions, and aggregation state in the presence of analytes, AuNPs possess a unique colorimetric feature that makes them an ideal platform for biosensing [118]. A discernible shift from red to blue is caused by the transition of GNPs in a liquid medium, going from the monodisperse to the aggregated state. Nanoparticles possess elevated surface energy and a substantial surface-to-volume ratio, with diameters ranging from 1 to 100 nm. Their capacity to directly transport electrons between various electroactive substances and electrodes is remarkable. Therefore, AuNPs are used in the production of optical, electrochemical, and piezoelectric biosensors for biosensing purposes. The correct signal is provided by optical biosensors by sensing changes in the photon output, or light. Optical biosensing makes use of AuNPs because of their surface plasmon resonance (SPR) activity and optical sensing modality. It is well-known that AuNPs may amplify SPR signals, which in turn can increase the local electro-magnetic field, which in turn can generate luminescence from the surface of a metal-liquid [119]. The capacity of biosensors to identify a diverse array of allergens and pollutants has relied heavily on these unique AuNPs properties for food safety applications.

**Figure 7:**
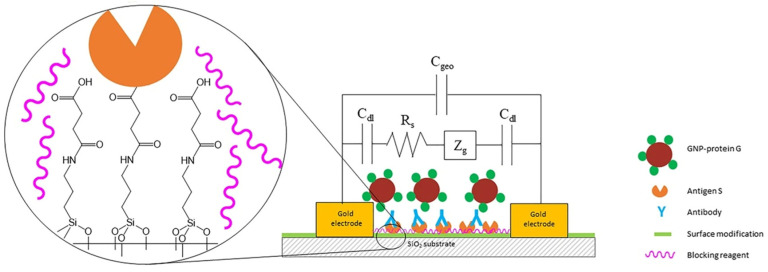
Affinity biosensor schematic diagram. The space between interdigitated electrodes immobilizes the pure S protein (antigen S) of the baculovirus. The S protein is bound by serum antibodies to it. An impedance change results from the binding of AuNPs coupled with protein G (AuNPs -protein G) to the antibodies. The silica gap between Au electrodes with surface modification details, spike protein immobilization, and the presence of blocking agents to enhance impedance measurements are all visible in the zoomed-in region. Reproduced [120] with the permission of the Creative Commons Attribution 4.0 International License (http://creativecommons.org/licenses/by/4.0/ (accessed on 10 October 2025)). Copyright 2022, Nature.

Because COVID-19 waves are continuing to spread around the globe, there is a growing need for a biosensor that is portable, affordable, and easy to detect the immunological and infection status of a population. Khaniani et al. [120] present an impedance-based affinity biosensor that makes use of interdigitated electrode (IDE) arrays for the detection of SARS-CoV-2 antibodies in serum. Through the process of functionalizing the surface of the IDEs with a baculovirus-expressed and purified Spike (S) protein, they were able to produce the biosensor that is capable of binding anti-SARS-CoV-2 antibodies. AuNPs conjugated with protein G were used to detect binding antibodies (Figure 7). An ELISA assay demonstrated that the purified S protein preferentially bound a commercial supply of anti-SARS-CoV-2 antibodies, using horseradish peroxidase-protein G to detect bound IgG. Furthermore, the pure S protein was shown to bind anti-SARS-CoV-2 antibodies in the serum of individuals who tested positive for COVID-19 infections. Following that, the authors proved that the biosensor can distinguish anti-SARS-CoV-2 antibodies with a sensitivity of 72% in only two hours. Using AuNP-protein G, the biosensor exhibited greater impedance changes with serum that was positive for COVID-19, whereas any impedance changes that occurred with negative serum were either negligible or diminished. The results of this experiment indicated that the biosensor was able to differentiate between sera from individuals with COVID-19 and those without the disease. The ability to differentiate was further enhanced by the use of poly(vinyl alcohol) as a blocking agent.

### 3.2. Gold Nanoparticle Polymer Biosensors

**Figure 8:**
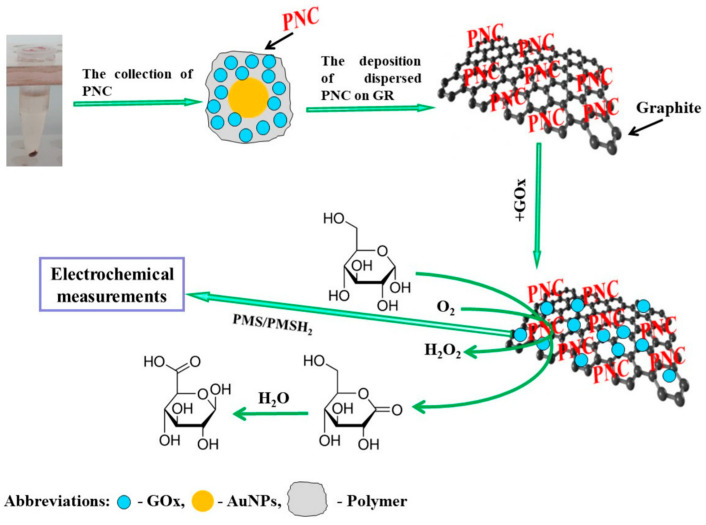
The deposition of PANI-AuNPs-GOx or PPy-AuNPs-GOx nanocomposites (PNC) on the GR electrode surface is shown schematically. Additional enrichment by GOx is performed after glucose is determined using the established enzymatic biosensors. Reproduced [121] with the permission of the Creative Commons Attribution 4.0 International License (http://creativecommons.org/licenses/by/4.0/ (accessed on 13 October 2025)). Copyright 2022, MDPI.

Biosensors capable of measuring glucose concentration hold significant importance in clinical diagnostics, as well as in the food and pharmaceutical industries. In the research developed by German et al. [121], nanocomposites containing GOx and 6 nm diameter AuNPs (AuNPs_(6 nm)_) were immobilized on graphite rod (GR) electrodes, after short-chain polyaniline (PANI) and PPy-based materials were implanted onto them (Figure 8). As a redox mediator, phenazine methosulfate was used to study the electrodes using CV and constant potential amperometry. The enhanced enzymatic biosensors that utilized GR/PANI-AuNPs_(6 nm)_-GOx/GOx and GR/PPy-AuNPs_(6 nm)_-GOx/GOx electrodes exhibited several desirable properties, such as a high sensitivity (65.4 and 55.4 μA mM^−1^ cm^−2^), a low LOD (0.070 and 0.071 mM, respectively), a wide linear range (up to 16.5 mM), good repeatability (RSD 4.67 and 5.89%), and suitable stability (half-life period, τ^1/2^, of 22 and 17 days, respectively). The GR/PANI-AuNPs_(6 nm)_-GOx/GOx electrode demonstrated good practical applicability for glucose detection in blood samples and demonstrated outstanding anti-interference capabilities against ascorbic and uric acids.

Assaying biomarkers in a way that is both rapid and selective while also being very sensitive is still a difficulty that is important to biosensor and diagnostic applications. This research by Ma et al. [122] presents the progress of a promising electrochemical biosensor that is based on three-dimensional nanoporous conducting polymer poly(3,4-ethylenedioxythiophene) (PEDOT) decorated with AuNPs. For the development of this biosensor, methylene blue was used as a redox indicator for the detection of microRNA24 (miRNA24). The one-of-a-kind porous architecture was created via the use of a straightforward hard-template technology and cyclic voltammetry (CV) as an electrochemical technique. An enhancement in the interface conductivity and the provision of additional active sites for load identification components were achieved by the electrodeposition of AuNPs onto the porous PEDOT surface. The porous biosensor that was created, which was based on conducting polymer PEDOT and AuNPs, had a greatly increased specific surface area (about 2.4 times) and a significantly improved sensitivity (about 2.1 times) in comparison to the biosensor that was based on planar PEDOT. In addition to achieving a satisfactory stability, excellent selectivity, and a low LOD (0.38 fM), the porous biosensor was able to accomplish a miRNA24 quantification range that extended from 1 fM to 10 nM. Biosensors built from porous conducting polymers adorned with AuNPs provide a promising approach to improving sensitivity via increasing the sensing surfaces’ electroactive surface area.

An indium tin oxide (ITO) substrate that was functionalized with AuNPs and an amino-functionalized thiophene polymer P(ThiAmn) multilayer was used by Aydin et al. [123] to create a novel immunosensor for impedimetric detection of GM2 activator protein (GM2A). For the purpose of engineering the biosensor, a rather straightforward method that included the electropolymerization of ThiAmn and the electrodeposition of GNPs was employed. Through the use of GNPs and P(ThiAmn), the surface area of the substrate was enhanced, which proved to be advantageous in terms of immobilizing a substantial quantity of anti-GM2A biorecognition components. The bifunctional layer that was created proved to be a very useful matrix material and allowed for the creation of novel sensors. To explore the particular immuno-interaction mechanism that occurs between biorecognition anti-GM2A antibodies and GM2A antigens, electrochemical methods were used. Spectral methods were also applied for the characterization of various modified electrode surfaces, in addition to these approaches. Under ideal circumstances, the EIS methodology was used to ascertain the concentration of GM2A in a linear concentration range that extended from 0.0185 to 111 pg mL^−1^. The LOD obtained was 5.8 fg mL^−1^. This biosensor demonstrated great specificity for GM2A antigens, as well as outstanding repeatability and extended storage stability. In addition, this immunosensor was used to measure GM2A in commercial blood samples, and the findings obtained were adequate.

On the surface of cancer cells that have developed from endodermal cells is a glycoprotein called carcinoembryonic antigen (CEA), which is an acidic glycoprotein with human embryonic antigen characteristics. In this research by Wang et al. [124], a label-free electrochemical immunoassay for the dual amplification detection of CEA is presented. The immunoassay makes use of AuNPs loaded with PPy polydopamine (Au/PPy-PDA) and polymerized polycaprolactone (Ng-PCL) that have been synthesized using ring-opening polymerization. Before anything else, the composite Au/PPy-PDA was affixed to the surface of the electrode. Following this, AuNPs establish an Au–S connection with the sulfhydryl group in Apt1 to firmly establish it on the surface of the electrode. Subsequently, bovine serum albumin (BSA) is used for closing the non-specific binding sites that are located on the surface of the electrodes. After that, CEA is dripped over the electrode surface, which is then immobilized via antigen–antibody specific recognition. The carboxyl-functionalized Apt2 then forms a “sandwich structure” consisting of antibody, antigen, and antibody by specific recognition. A full chain of signal analysis is achieved as a consequence of the adhesion of polymeric Ng-PCL to the electrode surface. This leads to a boost in the electrochemical impedance signal. In conclusion, EIS is used to successfully detect the response signal. When the experimental circumstances are appropriate, the approach has the benefits of high sensitivity and a broad concentration range of 1.0 × 10^−3^ to 1.0 × 10^2^ ng mL^−1^. Furthermore, the lower LOD is 0.234 pg mL^−1^. In addition to this, it has the same high sensitivity, selectivity, and interference resistance for the detection of genuine samples. Therefore, it offers a fresh perspective on the way biological and clinical diagnostics should be approached.

**Figure 9:**
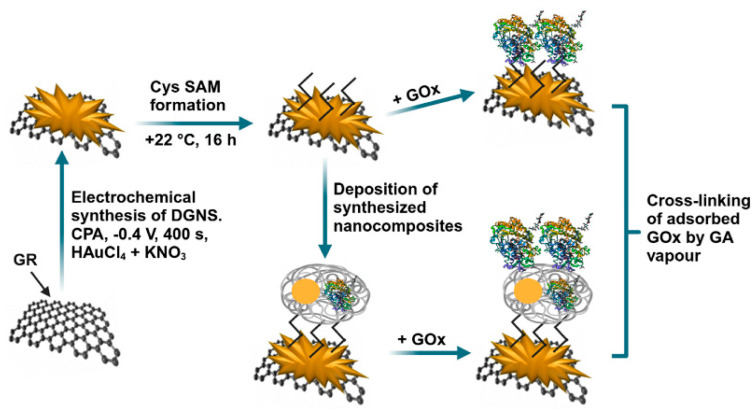
Glucose biosensors are schematically represented using GR/DGNS/Cys/GOx and GR/DGNS/Cys/PANI-AuNPs-GOx/GOx electrodes. Reproduced [125] with the permission of the Creative Commons Attribution 4.0 International License (http://creativecommons.org/licenses/by/4.0/ (accessed on 20 October 2025)). Copyright 2022, MDPI.

The medical condition known as diabetes mellitus necessitates the constant monitoring of blood glucose content. Two glucose biosensors that do not use enzymatic mediators and that use premodified graphite rod electrodes were created and evaluated by German et al. [125]. The electrochemical investigation focused on GR electrodes that had been modified with electrochemical synthesis of dendritic gold nanostructures (DGNS), a cystamine self-assembled monolayer (CysSAM), and GOx (GR/DGNS/Cys/GOx), as well as GR electrodes that had been modified with DGNS, Cys SAM, enzymatically obtained PANI nanocomposites with embedded 6 nm AuNPs and GOx (GR/DGNS/Cys/PANI-AuNPs-GOx/GOx) (Figure 9). The biosensors that utilized GR/DGNS/Cys/GOx and GR/DGNS/Cys/PANI-AuNPs-GOx/GOx demonstrated the following characteristics: a linear range of up to 1.0 mM of glucose; a storage stability of more than seventy-one days; a sensitivity of 93.7 and 72.0 μA mM^−1^ cm^−2^, respectively; a LOD of 0.027 and 0.034 mM; and, respectively, a reproducibility of 13.6 and 9.03% and a repeatability of 8.96 and 8.01%. For blood glucose concentration measurement, the GR/DGNS/Cys/PANI-AuNPs-GOx/GOx electrode was suggested as a superior option because of its improved stability and resistance to electrochemically active species that interfere. Innovative mediator-free enzymatic glucose biosensors, made possible by the technical solutions offered in this research, will improve clinical tests and help diabetics regulate their blood glucose levels.

Humans must be able to identify seroconversion to the coronavirus 2 (SARS-CoV-2) quickly and reliably for effective infection management. Much research has concentrated on improving sensitivity, decreasing detection limits, and reducing the number of false positives and negatives in this regard. For these reasons—a larger surface area, better electrical conductivity, and electrochemical activity—biosensors built on conducting polymer nanoarchitectures offer hope over more conventional materials. Hryniewicz et al. [126] demonstrated the analytical comparison of two distinct morphologies of conducting polymers to create an impedimetric biosensor to track human SARS-CoV-2 seroconversion. The biosensors have incorporated PPy and AuNPs for their design. These biosensors are made utilizing a self-assembly monolayer of 3-mercaptopropionic acid and covalently attached SARS-CoV-2 nucleocapsid protein, and they can be manufactured in both globular and nanotubular (NT) morphologies. The innovative hybrid materials were first studied using electron microscopy and electrochemical measurements; the biosensor’s sequential fabrication was then studied using electrochemical and spectroscopic methods. The biosensor was tested for its ability to detect anti-SARS-CoV-2 Nucleocapsid protein monoclonal antibodies by impedimetric analysis as proof. The findings demonstrated a steady relationship between antibody concentration and reaction, excellent sensitivity, and the ability to detect PPy at concentrations of 7.442 and 0.4 ng mL^−1^, respectively, in the globular and NT morphologies. The PPy-NTs biosensor, a potential tool for COVID-19 immunodiagnostics, successfully separated serum from positive and negative clinical samples, paving the way for further research on efficient, reliable, and fast detections. A summary of the investigations that have been performed on polymer biosensors with gold nanoparticles that can be used to find different biomarkers can be found in Table 7.

**Table 7:** Available research on gold nanoparticle polymer biosensors for biomarker detection.

Electrode	Linear Range	Detection Limit	Sensitivity	Refs.
GR/PANI-AuNPs_(6 nm)_-GOx/GOx	0.10–16.5 mM	0.070 mM	65.4 μA mM^−1^ cm^−2^	[121]
AuNPs/PEDOT/GCE	1 fM–10 nM	0.38 fM	-	[122]
GNPs-P(ThiAmn) multilayer coated ITO electrode	0.0185–111 pg mL^−1^	5.8 fg mL^−1^	0.212 kΩ pg^−1^ mL cm^−2^	[123]
Ng-PCL/Apt2/CEA/BSA/Apt1/Au/PPy-PDA	1.0 × 10^−3^–1.0 × 10^2^ ng mL^−1^	0.234 pg mL^−1^	-	[124]
GR/DGNS/Cys/GOx	0.050–1.0 mM	0.027 mM	93.7 μA mM^−1^ cm^−2^	[125]
GR/DGNS/Cys/PANI-AuNPs-GOx/GOx		0.034 mM	72.0 μA mM^−1^ cm^−2^	

## 4. Conclusions and Future Outlooks

Recent biosensor applications include microbiology, industrial process control, environmental monitoring, and food quality control. In vitro screening for early viral illness diagnosis has major medical implications for health monitoring and chronic disease treatment.

The physicochemical properties of nanocomposites determine biosensor performance. Because of their high conductivity and distinctive morphologies, CNTs are ideal for electrochemical and flexible wearable sensors. FET and MOF biosensors benefit from GR’s excellent conductivity and flexibility. Quantum dots should be employed for colorimetric or fluorescence responses on sensitive optical detection systems. GNPs are ideal for biosensing because their colorimetric property changes with size, shape, and analyte aggregation. Unique characteristics offered by modern polymer nanocomposite materials include a larger surface area for electrochemical activity, electrocatalytic activity towards specific analytes, and optical features that might be used in the development of innovative biosensors. Biosensors that detect electrochemical changes by means of metal nanocomposites, including Pt, Au, Ag, and Ni, increase sensitivity and decrease detection limits by enhancing electron transport between the analyte and electrode. These NPs’ large surface area and catalytic activity accelerate and improve redox processes. Metal nanocomposites may be functionalized with antibodies, enzymes, or DNA to improve biosensor specificity and sensitivity. Nanoparticles’ high surface area enables immobilization of several biorecognition components, enhancing target binding. Metal nanocomposites enhance biosensor stability and reusability by being a reliable platform for immobilizing biorecognition elements. These materials demonstrate chemical and mechanical stability, ensuring long-term performance in hostile conditions.

Nanocomposites are a great way to increase the sensitivity and active surface area of a sensor. They are made by mixing nanomaterials to generate porous structures with a high surface-to-volume ratio, which increases the number of sites for analyte interaction. Increased surface area provides more active areas for analyte interactions, improving biosensor sensitivity and lowering detection limits.

In the future, biosensors might transform healthcare, diagnosis, and health monitoring. Connecting biosensors to smartphones will revolutionize health monitoring by measuring several elements from anywhere. Doctors will have immediate access to patient data in conjunction with cloud-based diagnostics, allowing for speedier and more accurate treatment decisions. Fully biodegradable biosensors may also alleviate biological waste concerns. These single-use, eco-friendly devices will replace traditional diagnostic tools and promote green healthcare waste management.

## Data Availability

No new data were created or analyzed in this study. Data sharing does not apply to this article.
